# Plant Diversity and Microbial Community Drive Ecosystem Multifunctionality in *Castanopsis hystrix* Plantations

**DOI:** 10.3390/plants14131973

**Published:** 2025-06-27

**Authors:** Han Sheng, Babar Shahzad, Fengling Long, Fasih Ullah Haider, Xu Li, Lihua Xian, Cheng Huang, Yuhua Ma, Hui Li

**Affiliations:** 1 College of Forestry & Landscape Architecture, South China Agricultural University, Guangzhou 510642, China; shenghan@stu.scau.edu.cn (H.S.); fllong@scau.edu.cn (F.L.); xianlihua@scau.edu.cn (L.X.); 2 Guangdong Provincial Key Laboratory of Applied Botany, South China Botanical Garden, Chinese Academy of Sciences, Guangzhou 510650, China; haider281@scbg.ac.cn (F.U.H.); lixu@scbg.ac.cn (X.L.); 3 Ecological Plant and Animal Sciences, La Trobe University, Melbourne, VIC 3086, Australia; b.shahzad@latrobe.edu.au; 4 Hubei Key Laboratory of Biologic Resources Protection and Utilization, Hubei Minzu University, Enshi 445000, China; 2024102@hbmzu.edu.cn; 5 College of Civil and Architecture Engineering, Chuzhou University, Chuzhou 239099, China; myh@chzu.edu.cn

**Keywords:** biodiversity, ecological restoration, ecosystem services, forest ecosystem, stand age

## Abstract

Monoculture plantation systems face increasing challenges in sustaining ecosystem multifunctionality (EMF) under intensive management and climate change, with long-term functional trajectories remaining poorly understood. Although biodiversity–EMF relationships are well-documented in natural forests, the drivers of multifunctionality in managed plantations, particularly age-dependent dynamics, require further investigation. This study examines how stand development influences EMF in *Castanopsis hystrix* L. plantations, a dominant subtropical timber species in China, by assessing six ecosystem functions (carbon stocks, wood production, nutrient cycling, decomposition, symbiosis, and water regulation) of six forest ages (6, 10, 15, 25, 30, and 34 years). The results demonstrate substantial age-dependent functional enhancement, with carbon stocks and wood production increasing by 467% and 2016% in mature stand (34 year) relative to younger stand (6 year). Nutrient cycling and water regulation showed intermediate gains (6% and 23%). Structural equation modeling identified plant diversity and microbial community composition as direct primary drivers. Tree biomass profiles emerged as the strongest biological predictors of EMF (*p* < 0.01), exceeding abiotic factors. These findings highlight that *C. hystrix* plantations can achieve high multifunctionality through stand maturation facilitated by synergistic interactions between plants and microbes. Conservation of understory vegetation and soil biodiversity represents a critical strategy for sustaining EMF, providing a science-based framework for climate-resilient plantation management in subtropical regions.

## 1. Introduction

Forests are a crucial part of terrestrial ecosystems, providing essential services such as carbon sequestration, oxygen production, hydrological regulation, and soil stabilization [1,2]. However, these services are threatened by intensifying anthropogenic pressures, such as deforestation, land-use change, and climate change, which are leading to more frequent and severe weather events. These factors drive the widespread fragmentation of forest ecosystems and the decline in ecological functions [3]. Addressing these challenges through forest conservation and restoration has become an international priority, particularly in tropical and subtropical regions where degraded and regenerating forests create complex, dynamic interactions between ecosystem structure and functionality [4]. These vulnerabilities not only threaten carbon storage capacity but also compromise the ability of forests to sustain essential human demands [5,6]. Understanding multifunctionality in these ecosystems is thus critical for developing adaptive management strategies that align with shifting climatic conditions and societal requirements.

The concept of ecosystem multifunctionality (EMF) refers to an ecosystem’s ability to provide multiple ecological functions and services simultaneously. This has become a key area of research in recent years [5,7,8]. While studies into the effects of stand age on EMF have mainly focused on secondary forest succession [4,9,10], the passage of stand age is a fundamental driver of multifunctional dynamics [4,10,11]. Research has shown that tree diversity is positively linked to EMF, with mixed-species systems generally performing better than monocultures in providing ecological services [3,6,12]. However, there remains significant uncertainty regarding the EMF potential of intensively managed monoculture plantations compared to natural forests or diversified stands, particularly under long-term single-species cultivation regimes.

Current research priorities in forest ecosystems emphasize analyses of soil nutrient dynamics, vegetative productivity, and biodiversity metrics [13,14], while comprehensive evaluations of plantation ecosystem functionality remain comparatively understudied. Traditional ecosystem service assessments face methodological challenges, as differential valuation by stakeholders often introduces subjective weighting schemes that may bias results [15]. This limitation prompted the development of EMF as an integrative framework, conceptualizing an ecosystem’s capacity to deliver multiple functions and services concurrently [16,17,18]. These functions represent foundational ecological processes that underpin service provision [9,10]. Thus, the EMF paradigm offers theoretical foundations and analytical tools to assess and enhance functional diversity in plantation systems, supporting more sustainable forest management approaches.

Recent scholarship has increasingly recognized plantation forests as complex systems capable of delivering multiple ecosystem services simultaneously [5,16,19]. Temporal dynamics represent a critical dimension in understanding EMF [4,20]. While numerous investigations have examined age-related patterns in forest EMF [4,9,20], with consistent observations of functional enhancement in mature stands [5,9,20,21], the underlying processes remain poorly understood. Current evidence suggests that both edaphic and plant diversity serve as primary determinants of EMF variation [9,21], with global-scale analyses confirming significant associations between soil biodiversity and multifunctional performance [22]. Nevertheless, the specific pathways through which factors mediate EMF in managed plantations require further elucidation.

Within China’s southern subtropical region, *Castanopsis hystrix* has emerged as the predominant coniferous species for large-scale timber production [19,23,24,25]. While meeting growing wood demands, intensive *C. hystrix* monocultures have generated ecological concerns, including marked soil deterioration and reduced biodiversity [19,23,24,25]. Such intensive silvicultural approaches may compromise the long-term functionality of ecosystems. Hence, this study aimed to investigate the EMF across varying stand ages in *C. hystrix* plantations, identify the principal factors governing EMF patterns in different stand-age forests, and establish an empirical foundation for optimizing plantation management strategies in these ecoregions.

## 2. Materials and Methods

### 2.1. Study Area

The research was conducted at the Longyandong Forest Farm in Guangdong Province (23°10′–23°18′ N, 113°21′–113°27′ E), characterized by tropical monsoon conditions, with a mean annual temperature of 21 °C, 1900 mm of precipitation, and 80% relative humidity. The predominant lateritic red soils exhibit vertical profiles with depths of 30–100 cm and acidic pH values (4.5–6.5). Study plantations were established through even-aged regeneration following clear-cutting in 1986, 1990, 1995, 2005, 2010, and 2014. Uniform silvicultural protocols, including standardized fertilization and cultivation practices, were applied across all sites, with anthropogenic interventions ceasing between years 3 and 5 post-establishment [23,24,25]. Historical land-use patterns and geological conditions were consistent across all plantation sites before the introduction of *C. hystrix*.

### 2.2. Plot Design and Soil Sampling

Field investigations were conducted during August 2020 across six representative stands, each containing three randomly positioned 400 m^2^ monitoring plots (eighteen total sampling units). Rigorous site selection criteria [26] ensured that management history represented the primary variable: (1) homogeneous edaphic and topographic conditions; (2) shared land-use legacy as second-generation plantations established on former *Acacia mangium* sites converted from natural forests during the 1970s; (3) comparable developmental stages (6–34 years) with analogous initial species composition; and (4) standardized management regimes implemented by a single forestry collective [23,24,25]. Comprehensive biodiversity assessments documented all vascular plants across vertical strata, recording species identities, diameter at breast height (DBH; ±0.1 cm precision), and height (±0.5 m) for subsequent stand basal area calculations. Soil profiles were systematically sampled across three depth intervals (0–10, 10–20, and 20–30 cm) using stainless steel augers (4 cm diameter), with three replicates per depth. Seven spatially distributed subsamples per depth were homogenized into composite specimens [23,24,25,27]. All samples were immediately preserved at 4 °C in dark conditions to maintain biogeochemical integrity.

### 2.3. Sampling Analysis

Soil biogeochemical properties were comprehensively characterized through a suite of analytical measurements. Soil organic carbon (SOC) was measured by oxidation with potassium dichromate and external heating [16,23]. Total nitrogen (TN) content was estimated through the Kjeldahl method after digestion [24]. Total phosphorus (TP) content was assessed using a photometer [24]. Physical parameters, including soil water content and pH, were determined for all samples. Extracellular enzyme activities were assessed for decomposition function [19]: acid phosphomonoesterase (AP), β-glucosidase (BG), N-acetylglucosaminidase (NAG), cellobiohydrolase (CBH), and phenol oxidase (PhOx). The procedure was as follows: 2.0 g of fresh soil sample was added to 30 mL of acetate buffer and stirred for 1 min, and then 0.75 mL of the mixture was aspirated to a 96-deep well plate, followed by 0.75 mL of substrate added to the mixture in the 96-deep well plate. The absorbance values of hydrolytic enzymes (including AP, BG, CBH, and NAG) and oxidative enzymes (including POD and PPO) were measured at 405 nm and 450 nm, respectively, using an enzyme marker (Thermo Scientific Multiskan) [19,24].

Microbial biomass carbon (MBC) and nitrogen (MBN) were determined as follows: two 10 g fresh soil samples were weighed into two 100 mL glass vials and placed in the vacuum desiccator fumigated with chloroform and without fumigation, respectively, for 24 h. Then, 30 mL of 0.5 M K_2_SO_4_ was added to the two vials, and the vials were shaken at a medium speed for 1 h and then filtered to obtain the supernatant for determination. Microbial community composition, including total phospholipid fatty acids (total PLFAs), bacteria, fungi, Gram-positive bacteria (G^+^ bacteria), Gram-negative bacteria (G^−^ bacteria), actinomycetes (Act), arbuscular mycorrhizal fungi (AMF), and ectomycorrhizal fungi (EMF), was profiled through phospholipid fatty acid (PLFA) analysis. This process includes the extraction, separation, and purification of phospholipids from soil extracts, followed by the addition of methanol to facilitate the reaction that forms fatty acid methyl esters. The content of various fatty acids is then determined by chromatography. All analytical procedures were conducted using rigorously validated protocols [16,24], with quality assurance measures including certified reference materials, method blanks, and analytical replicates to ensure data reliability.

### 2.4. Evaluating Ecosystem Multifunctionality

Six key ecosystem functions, including carbon stocks, nutrient cycling, wood production, decomposition, symbiosis, and water regulation, were evaluated as integrated proxies for ecosystem multifunctionality (EMF) [4,9,16]. For quantitative assessment, each functional metric was first normalized to a 0–1 scale to account for variable dimensional differences. The composite EMF index was then derived as the arithmetic mean of these standardized values [28], representing overall ecosystem performance. Complete operational definitions and measurement protocols for all functional indicators are systematically presented in Table S1.

### 2.5. Statistical Analysis

The analytical approach integrated multiple statistical paradigms to comprehensively evaluate ecosystem dynamics. Treatment effects across standard age classes were rigorously assessed using one-way ANOVAs, complemented by Duncan’s post hoc tests, to ensure the robust detection of individual ecosystem functions and composite multifunctionality. Interdependencies between environmental covariates and functional metrics were examined through two approaches: Pearson correlation analysis for linear associations and the Mantel Test for distance-based relationships, which were executed using specialized R packages (*linkET* and *tidyverse*). Predictive modeling, incorporating machine learning (Random Forest via *randomForest* and *rfPermute* packages), identified key drivers, while structural equation modeling (*piecewiseSEM*) elucidated the complex interaction networks governing EMF. Biodiversity quantification employed five complementary indices (species richness, Simpson’s dominance, Shannon–Wiener diversity, Pielou’s evenness, and Margalef’s richness) processed through the *vegan* and *reshape2* packages. All analyses were conducted in R version 4.4.2 with stringent diagnostic validation, while visualization leveraged *ggplot2* and GraphPad Prism version 9.0 for publication-quality graphical representation.

## 3. Results

### 3.1. Individual Ecosystem Functions and Multifunctionality

**Figure 1:**
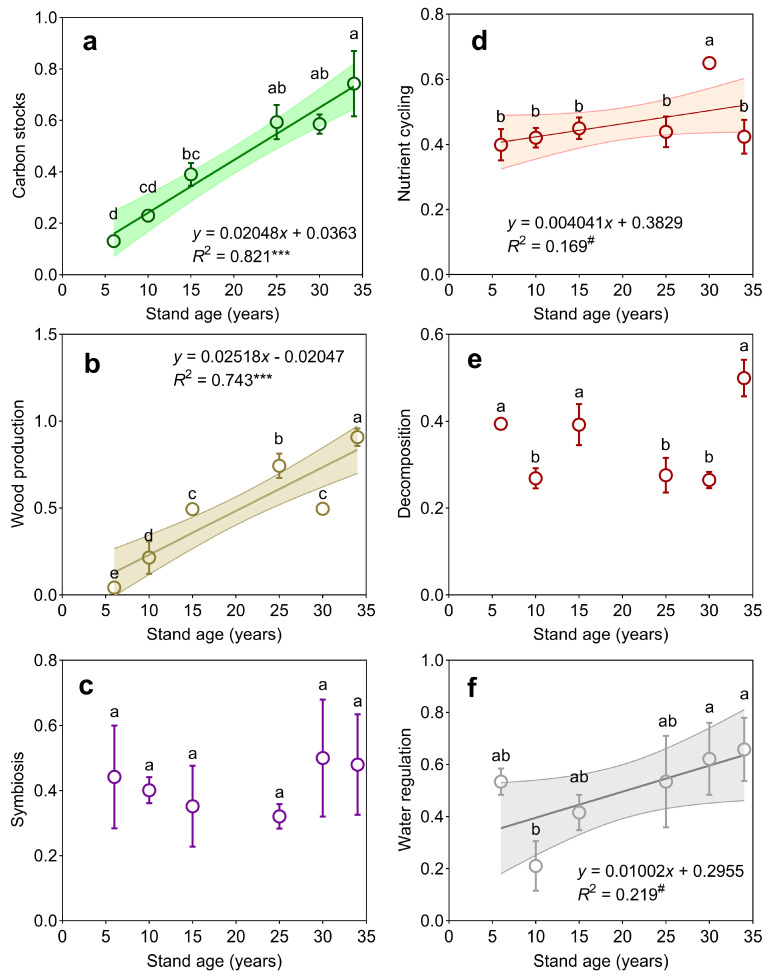
Ecosystem functions of *Castanopsis hystrix* plantation at different stand ages: (**a**) carbon stocks, (**b**) wood production, (**c**) symbiosis, (**d**) nutrient cycling, (**e**) decomposition, and (**f**) water regulation. Values represent mean ± standard error (n = 3). Lowercase letters denote significant pairwise differences (Tukey’s HSD, α = 0.05). Correlation significance levels: *** *p* < 0.001; ^#^ *p* < 0.10 (trend level).

**Figure 2:**
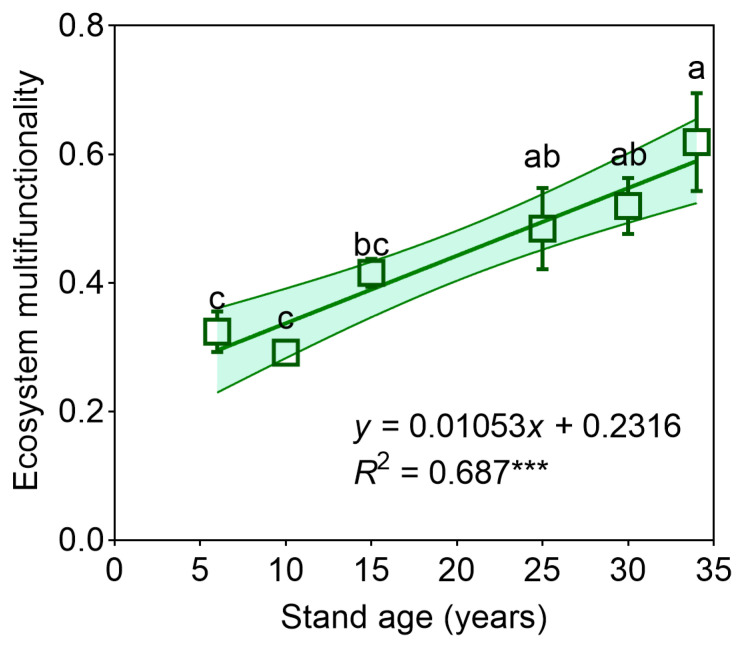
Ecosystem multifunctionality (EMF) of *Castanopsis hystrix* plantation at different stand ages. Error bars represent the standard error of the mean. Distinct superscript letters (a, b, and c) reflect significant divergence between age classes (one-way ANOVA, Tukey post hoc, α = 0.05). Asterisks denote statistical significance: *** *p* < 0.001.

Stand age exerted significant positive effects on carbon stocks (*p* < 0.001) and wood production (*p* < 0.001), with strong linear relationships evident between stand age and these functional metrics (Figure 1a,b). Marginal yet consistent age-dependent enhancements emerged for nutrient cycling function (*p* < 0.10) and water regulation capacity (*p* < 0.10; Figure 1d,f). In contrast, symbiosis and decomposition functions remained invariant across the chronosequence (Figure 1c,e). Composite EMF exhibited progressive amplification with stand maturation (Figure 2), demonstrating synchronous enhancement of multiple critical functions.

### 3.2. Relationships Among Ecosystem Functions

**Figure 3:**
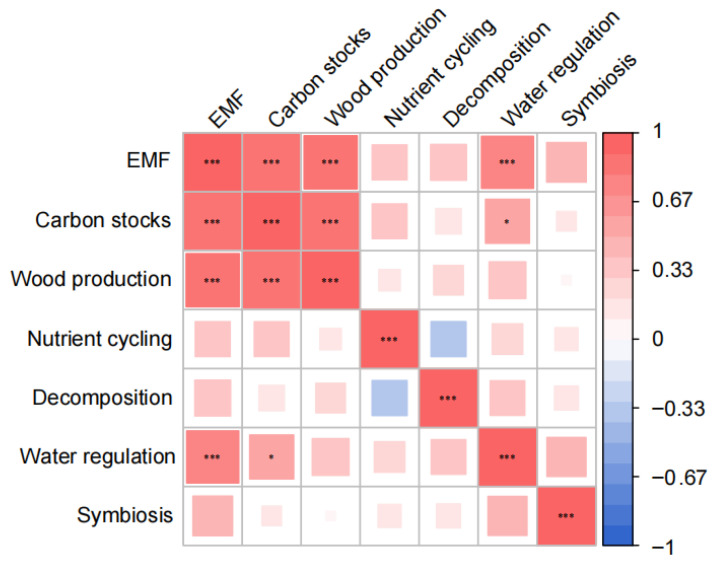
Functional interdependencies among key ecosystem processes in *Castanopsis hystrix* plantations. Correlation strength is shown with asterisks denoting statistical significance: * *p* < 0.05; *** *p* < 0.001.

The results uncovered a strong coupling between key ecosystem processes (Figure 3). The composite EMF demonstrated particularly robust associations with both the carbon stock function (*p* < 0.001) and the wood production function (*p* < 0.001). Furthermore, water regulation performance showed a significant positive linkage with carbon stock function (*p* < 0.05), revealing synergistic interactions between these functional domains.

### 3.3. Factors Influencing Individual Ecosystem Functions and Multifunctionality

**Figure 4:**
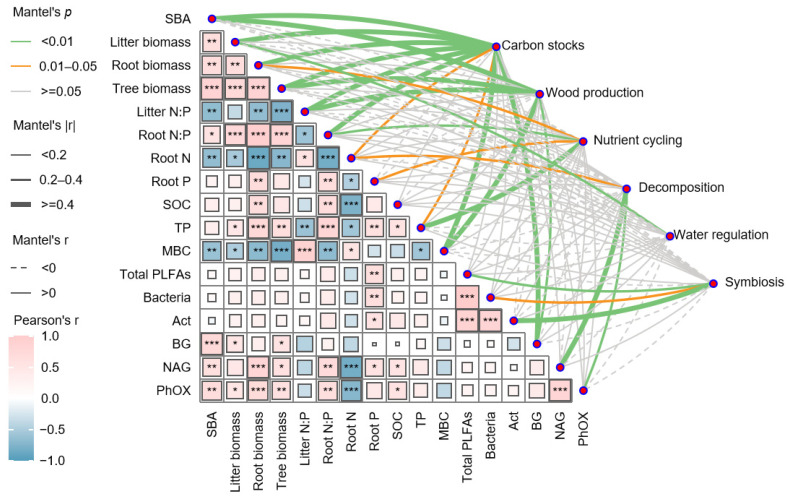
Relationships between edaphic/vegetation characteristics and ecosystem functional components (carbon stocks, wood production, symbiosis, nutrient cycling, decomposition, and water regulation) of *Castanopsis hystrix* L. at different stand ages. Significance thresholds: * *p* < 0.05, ** *p* < 0.01, and *** *p* < 0.001. Abbreviations: SBA (stand basal area), SOC (soil organic carbon), TN (total nitrogen), MBC (microbial biomass C), PLFAs (phospholipid fatty acids), Act (actinomycetes), BG (β-glucosidase), NAG (N-acetylglucosaminidase), and PhOx (phenol oxidase).

Correlation analysis further revealed a strong coupling between SOC and TP concentrations (*p* < 0.05), with parallel associations observed between SOC and both NAG and PhOX activities. Root phosphorus content showed positive linkages with TP, total phospholipid fatty acids (total PLFAs), and bacterial abundance (*p* < 0.01). BG activity covaried significantly with stand structural parameters, including basal area (SBA), litter biomass, and tree biomass (*p* < 0.05). Similar patterns emerged for NAG and PhOX activities, which were positively associated with tree and root biomass (Figure 4).

Mantel test identified robust multivariate relationships between ecosystem functions and their putative drivers. Carbon storage capacity demonstrated significant associations (r ≥ 0.4, *p* < 0.05) with stand structure (SBA and tree biomass), detrital inputs (litter biomass), root traits (biomass and N:P ratio), and microbial activity (MBC and BG). The wood production function exhibited analogous dependencies on SBA and tree biomass, while nutrient cycling was primarily governed by root phosphorus content and total phosphorus (TP) availability. The decomposition function was strongly correlated with root nitrogen content, NAG, and PhOX activities, whereas litter accumulation predominantly influenced water regulation. Symbiosis function exhibited direct relationships with total PLFAs and actinobacterial abundance (*p* < 0.05).

**Figure 5:**
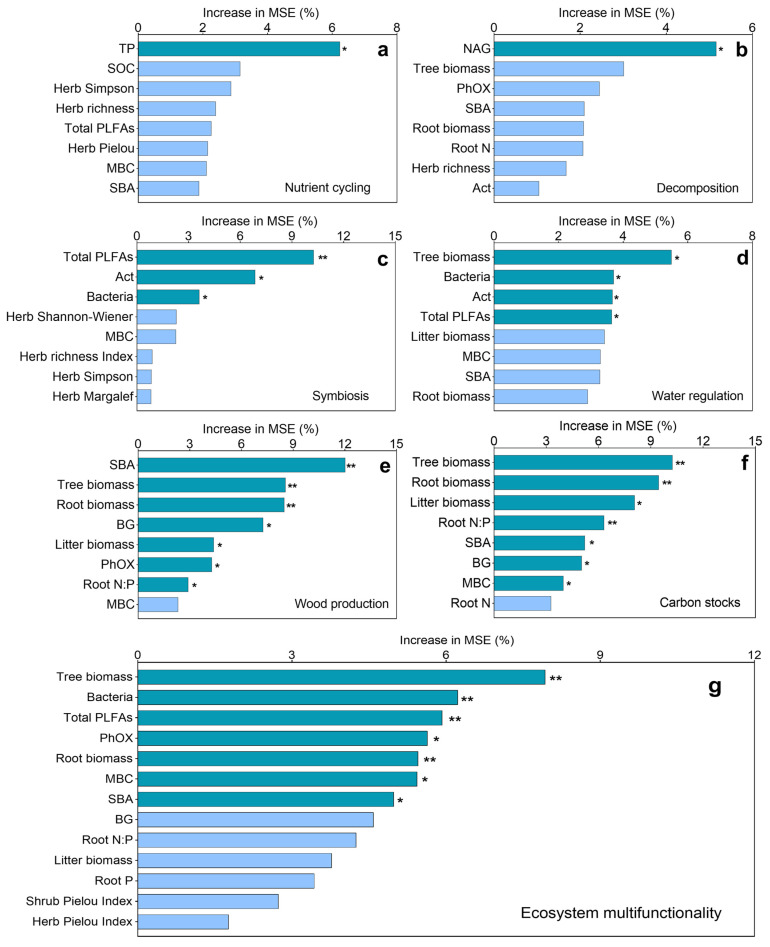
Key drivers of (**a**) nutrient cycling, (**b**) decomposition, (**c**) symbiosis, (**d**) water regulation, (**e**) wood production, (**f**) carbon stocks, and (**g**) ecosystem multifunctionality in *Castanopsis hystrix* plantations identified through random forest modeling. The predictive importance of edaphic and biotic factors is shown for six functional components. Variable importance is quantified by the percentage increase in mean square error (MSE), with higher values indicating greater predictive power. Significance levels: * *p* < 0.05 and ** *p* < 0.01. Abbreviations: PLFAs (phospholipid fatty acids), SBA (stand basal area), PhOx (phenol oxidase), MBC (microbial biomass carbon), and BG (β-glucosidase).

Random forest modeling analysis (Figure 5) revealed a hierarchical control of ecosystem processes, with soil microbial communities (total PLFAs and Act) emerging as primary regulators of symbiotic functions. TP content was the dominant constraint on nutrient cycling, while NAG activity mediated decomposition pathways. Stand structural components (tree biomass and SBA) and root traits collectively shaped wood production and carbon sequestration. Notably, ecosystem multifunctionality was jointly governed by vegetation characteristics (tree biomass and root biomass), microbial parameters (bacteria, total PLFAs, and MBC), and extracellular enzyme activities (PhOX), highlighting the integrated nature of functional regulation in these plantation systems.

### 3.4. Contribution of Plant Diversity and Microbial Characteristics to Ecosystem Multifunctionality

**Figure 6:**
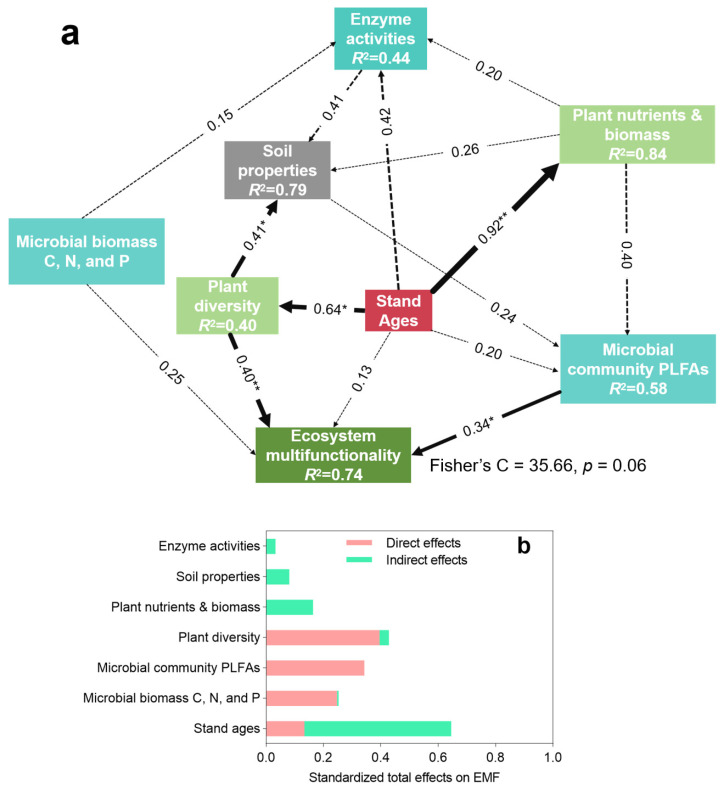
(**a**) Structural equation modeling of stand ages, vegetation characteristics, soil parameters, and microbial community composition (PLFAs). (**b**) effects on ecosystem multifunctionality (EMF) in *Castanopsis hystrix* plantations. Path coefficients denote standardized effect sizes: * *p* < 0.05 and ** *p* < 0.01.

Structural equation modeling elucidated the hierarchical effects of environmental factors on EMF in *C. hystrix* plantations (Figure 6). Stand age exhibited strong positive linkages with both plant nutrient status and biomass (*p* < 0.01) and plant diversity (*p* < 0.05), which in turn directly influenced soil biogeochemistry (*p* < 0.05) and EMF (*p* < 0.01). Microbial community composition (PLFAs) demonstrated intermediate regulatory effects (*p* < 0.05), while soil properties and enzyme activities showed minimal contributions. The standardized total effects (Figure 6b) reveal a cascading control hierarchy: stand age > plant diversity > microbial communities > nutrient pools > soil properties > enzymatic processes. Notably, plant diversity and microbial parameters emerged as the dominant direct drivers of EMF.

## 4. Discussion

### 4.1. Shifts in Individual Functions and Ecosystem Multifunctionality with Forest Stand Age

Plantation forests contribute significantly to various ecosystem services [4,29]. The results of this study partially corroborate the hypothesis that both carbon storage and timber yield tend to rise as forest stands mature (Figure 1a,b), aligning well with earlier research findings. For instance, previous investigations have documented increased ecosystem carbon stocks and tree biomass with increasing stand age [27]. This enhancement in carbon accumulation with advancing stand age may partly be attributed to the elevated wood growth and litterfall associated with older forest stages [27,30,31]. Therefore, establishing and restoring *C. hystrix* plantations could boost carbon capture, mitigate climate change impacts, and enhance other ecosystem functions, thereby supporting future carbon reduction goals [16,19,27].

Regarding water regulation, this function exhibits an upward trend with increasing stand age (Figure 1f). In mature forest ecosystems, the complexity of both structure and function influences precipitation dynamics through mechanisms such as canopy interception, throughflow, and stemflow [32]. Additionally, changes in defoliation patterns linked to stand age may affect water movement within plantation forests. A positive correlation between water regulation and litter biomass suggests that abundant leaf litterfall can intercept surface runoff and rainfall more effectively, improving water retention as forests age [16]. Early-stage forests may demand more water due to rapid growth [33]. Nutrient cycling functions peak in younger stands (T6–T15 and T25–T30 years), likely driven by vigorous nutrient uptake by *C. hystrix*, but decline in older stands (T30–T34 years) as litter input and nutrient turnover slow down. Moreover, strong positive associations between carbon storage and wood production functions, supported by random forest modeling, highlight the importance of stand basal area (SBA), tree biomass, root biomass, and litter biomass as key determinants of these ecosystem services. This implies that cultivating large-diameter timber can effectively enhance carbon sequestration and wood productivity in forest ecosystems [19].

Stand age emerges as a critical factor shaping the functional attributes of forest ecosystems [9]. Our findings demonstrate a significant influence of stand age on EMF. Specifically, EMF tends to increase with stand maturity, a pattern supported by multiple studies [34,35,36]. Stand age, therefore, is a pivotal driver of EMF (Figure 2). Generally, older stands (T30–T34 years) exhibit superior EMF levels compared to younger cohorts (T6–T15 and T25–T30 years), particularly excelling in carbon sequestration and wood production capacities, reinforcing our initial premise. Structural equation modeling further revealed that EMF has a positive correlation with plant diversity within *C. hystrix* plantations [16,37]. Enhanced plant community diversity is also observed in the soils of older forests [24,38]. Plant diversity, plant nutrients, biomass, and EMF exhibit an increasing trajectory with stand age (Figure 6), underscoring the ecological value of conserving mature forest stands [1,9,27]. Typically, old-growth forests possess greater biodiversity, adaptability, and resilience compared to younger stands, translating into broader EMF benefits and supporting sustainable plantation forest management [4].

### 4.2. Plant Diversity and Microbial Communities Drive Ecosystem Multifunctionality

Soil biodiversity is critical in sustaining multiple ecosystem functions simultaneously, including EMF [4,38]. Our findings underscore that soil microbial communities are the predominant direct drivers of variation in EMF within *C. hystrix* plantations (Figure 6). Specifically, random forest analyses identified total PLFAs and bacteria as key predictors of EMF performance [4,39]. Complementing this, structural equation modeling revealed a robust direct linkage between soil microbial populations and forest EMF. This supports random forest modeling that distinct soil microbial taxa establish diverse associations with symbiotic functions and overall ecosystem multifunctionality. This phenomenon likely arises because different microbial groups occupy unique ecological niches, enhancing multiple ecosystem processes [31,40].

Vegetation diversity emerges as the principal biotic factor influencing fluctuations in forest EMF [9,41]. Correspondingly, stand age positively influences plant diversity, implying that the role of plant diversity in ecosystem functioning intensifies as forests mature [42,43]. Extensive empirical and observational studies have documented strong connections between plant communities and ecosystem services and functions [5,19,28]. Plant species richness, in particular, is recognized as a major driver of EMF [44]. In the context of our study, plant diversity was found to have a direct and significant effect on EMF within *C. hystrix* plantations (Figure 6). This effect may be attributed to increased plant diversity, which enhances resource heterogeneity, augments habitat structural complexity, and promotes the abundance and activity of soil microbial communities, all collectively bolstering ecosystem functions [22,45]. Therefore, preserving plant diversity and maintaining sufficient biomass in forest ecosystems are fundamental strategies for supporting and enhancing EMF.

Overall, EMF exhibited strong associations with plant diversity and microbial community characteristics, with plant diversity exerting a particularly pronounced influence on multifunctionality [19,31,46]. Our results further revealed that tree biomass was the most significant contributor to EMF, likely because biomass accumulation directly affects the ecosystem’s capacity for carbon sequestration. Additionally, greater tree biomass increases the input of organic materials into the soil, stimulating decomposition and nutrient transport processes, thereby enhancing ecosystem functions related to decomposition and nutrient cycling [38,47]. Furthermore, we observed a significant positive correlation between microbial PLFAs and EMF. Consequently, this study advocates for the retention of understory vegetation and the judicious application of nitrogen fertilizers as practical measures to improve ecosystem functioning in *C. hystrix* plantations and promote sustainable management [24].

### 4.3. Managing Forests to Drive Multiple Ecosystem Services and Limitations

Mounting evidence highlights the indispensable role of forests in delivering a wide array of ecosystem services, encompassing carbon sequestration, climate regulation [48,49], and biodiversity conservation [19,50]. The factors influencing EMF are inherently complex and multidimensional, necessitating studies such as ours to identify the principal indicators that exert significant control over EMF. Such insights are crucial for informing and optimizing forest management strategies. Our investigation demonstrated a clear positive relationship between stand age and EMF and further elucidated the key drivers of this pattern. Notably, as stand age increases, plant community diversity markedly increases, enhancing EMF. This finding underscores the importance of forest management approaches that promote species diversity, curb deforestation, augment understory vegetation, and implement three-dimensional planting designs to enrich vegetation community complexity and bolster forest ecosystem resilience [51]. These strategies offer valuable guidance for maximizing forest ecosystem functions.

Our results confirm that stand age exerts a significant influence on forest EMF. Forest ecosystems encompass a range of ecological functions, often exhibiting trade-offs among them [15,16,20]. Consequently, concentrating research efforts on a single ecosystem function risks neglecting others, which could lead to ecosystem degradation. EMF serves as an integrative metric that captures the overall functional performance of ecosystems [4,52], enabling a holistic framework for evaluating forest interventions and guiding management decisions. However, the diversity of EMF assessment methodologies, each with distinct strengths and limitations [1,15,53], poses challenges for cross-study comparisons and synthesis.

In this study, we adopted a comprehensive approach by integrating plant and soil attributes to characterize multiple ecosystem functions, including carbon storage, nutrient cycling, wood production, decomposition, water regulation, and symbiotic capacity. We then standardized these functional indicators to derive an aggregate EMF metric. While this methodology provides a robust and encompassing assessment of ecosystem multifunctionality, it does not remain easy to directly compare our EMF values with those reported in other studies due to variations in the selection of ecosystem function indicators. Therefore, we advocate for the research community to adopt a more unified framework that considers a broad spectrum of ecosystem functions and standardized characterization metrics, thereby enhancing comparability across studies. Future research efforts will further incorporate additional EMF evaluation techniques to improve the reliability and comparability of ecosystem multifunctionality assessments.

Moreover, our study was constrained by a relatively limited sample size, highlighting the need for expanded longitudinal investigations and larger-scale analyses to deepen our understanding of the long-term interplay between forest community structure and ecosystem service provision. Forest structure is dynamic and multifaceted, and the indicators selected here may not fully capture its complexity. Given the multidimensional and functionally intricate nature of forest ecosystem services, our current EMF measurement represents an initial yet incomplete portrayal. Future research will aim to expand the scope of EMF assessments by incorporating additional ecosystem services, such as forest air quality regulation and climate regulation, thereby enhancing the robustness and comparability of EMF evaluation methodologies. Furthermore, other factors that may influence EMF, such as site history, climate change, or plantation management practices, will continue to be explored.

## 5. Conclusions

This investigation demonstrates a consistent age-dependent enhancement of critical ecosystem functions, including carbon stocks (25–467%), wood production (22–2016%), nutrient cycling, and water regulation, across the development stages of *C. hystrix* plantations. Ecosystem multifunctionality (EMF) exhibited progressive amplification with stand maturation (*p* < 0.001), reflecting coordinated and linear functional synergies. Plant diversity and microbial parameters emerged as the dominant direct drivers of EMF. Notably, tree biomass signatures emerged as the most robust biological predictors of multifunctionality (*p* < 0.01), surpassing purely abiotic factors. These findings establish an empirical foundation for silvicultural strategies that simultaneously maintain soil biodiversity and structural complexity, thereby optimizing long-term ecosystem service provision in monoculture plantations.

## Data Availability

Data will be made available upon request.

## Supplementary Materials

The following supporting information can be downloaded at https://www.mdpi.com/article/10.3390/plants14131973/s1, Table S1: Operationalization of ecosystem multifunctionality through measurable functional attributes and their associated ecological indicators.
