# Supplementary material for: Plant Diversity and Microbial Community Drive Ecosystem Multifunctionality in Castanopsis hystrix Plantations
Source: Plants (Basel). 2025 Jun 27;14(13):1973. doi: 10.3390/plants14131973 (PMC12251890; doi:10.3390/plants14131973)
Supplement: Supplementary file 1 [file plants-14-01973-s001.zip › plants-3688920-supplementary.pdf]

plants

MDPI

Supplementary data

Table S1. Operationalization of ecosystem multifunctionality through measurable functional attributes and their associated ecological indicators.

Individual function	Indicator
Nutrient cycling	SOC, TN, NO_3^--N, NH_4^+-N, TP, and available P
Carbon stocks	Plant and soil carbon stock
Water regulation	Soil and litter water content
Wood production	Basal area and tree biomass
Decompositi on	Enzyme activities (AP, BG, NAG, CBH, PhOx, and Perox), Microbial biomass (MBC, MBN, and MBP)
Symbiosis	The abundance of microbial (total PLFAs, fungi, bacteria, G^+ bacteria, G^- bacteria, AMF, and Act)

Note: SOC, soil organic carbon; TN, total nitrogen; NO_3^--N, nitrate nitrogen; NH_4^+-N, ammonium nitrogen; TP, total phosphorus; available P, available phosphorus; AP, acid phosphomonoesterase; BG, β-glucosidase; NAG, nacetylglucosaminidase; CBH, cellobiohydrolase; PhOx, phenol oxidase; Perox, peroxidase; MBC, microbial biomass carbon; MBN, microbial biomass nitrogen; MBP, microbial biomass phosphorus; G^+ bacteria, gram-positive bacteria; G^- bacteria, gram-negative bacteria; AMF, arbuscular mycorrhizal fungi; and Act, actinomycetes.

 https://doi.org/10.3390/plants14131973
